# Pediatric Intussusception Following COVID-19 Infection: A Rare Presentation

**DOI:** 10.7759/cureus.23488

**Published:** 2022-03-25

**Authors:** Cuong D Tran, Christina Cheung, Benjamin Archambeau, Fanglong Dong, Michael M Neeki

**Affiliations:** 1 Emergency Medicine, Arrowhead Regional Medical Center, Colton, USA

**Keywords:** pediatric intussusception, ultrasound, computed tomography, emergency department, covid-19

## Abstract

Pediatric intussusception has been reported to be associated with coronavirus disease 2019 (COVID-19) infection in the literature since the start of the pandemic in the past two years. Although this occurrence is exceptionally rare, rapid diagnosis based on recognition of gastrointestinal manifestations, clinical examination, and ultrasound confirmation can expedite appropriate care and prevent delayed complications. Intussusception is the most common cause of intestinal obstruction and acute abdomen in pediatric patients. Without prompt identification, the disease process can lead to necrosis, bowel perforation, shock, and, subsequently, multiorgan failure. Intussusception has previously been associated with viral upper respiratory infections, which can cause mesenteric lymphadenopathy as a lead point to allow the bowel to telescope upon itself. The mechanism of how COVID-19 can contribute to intussusception without respiratory symptoms remains unknown. Here, we present a case of pediatric intussusception associated with COVID-19.

## Introduction

Intussusception is the most common cause of acute intestinal obstruction in pediatric populations [1]. Intussusception typically affects children up to 36 months of life; however, 60% of cases occur before the age of one [2]. Adult incidence represents 5% of total cases [3,4]. Intussusception is a gastrointestinal disease described by the invagination of a proximal segment of the intestine and its mesentery (intussusceptum) into a distal segment (intussuscipiens) [5]. This blocks the passage of food and restricts blood supply to the affected area, leading to bowel obstruction and ischemia. Although any tract of the mesentery can be involved, the largest proportion of cases (85%) is due to ileocolic involvement [5].

The most common initial presenting symptom of intussusception in children and infants is colicky abdominal pain [6]. Other symptoms include nausea and vomiting (usually bilious); additionally, children may feel shortness of breath, cry, and pull their knees to their chest [7]. This combination of vague and variable symptoms, often in an infant population, can make the clinical diagnosis of intussusception challenging. The “classic triad” of intussusception includes abdominal pain, vomiting, and bloody stool; however, historically, all three features may be noted in only 21% of cases [8]. More recent studies reported that the simultaneous presence of all three symptoms is seen in 46% of presentations [8,9]. Blood in stool is a part of the triad, but it is usually seen at a late stage, as this indicates necrosis has already occurred.

Once intussusception occurs, the mesentery is pulled along the leading aspect of the invaginated segment, and the mesenteric vessels of the intussusceptum are compressed leading to venous obstruction [10]. The invaginated segment becomes engorged resulting in bleeding from the mucosa, which combined with stool resembles bloody “red currant” stool [11]. If not diagnosed and treated promptly, it can potentially be a lethal condition. Prolonged intussusception compromises perfusion to the intestine and leads to necrosis of the bowel, perforation, and shock [11].

Coronavirus disease 2019 (COVID-19) can present as respiratory or non-respiratory symptoms. As discussed in Cai et al., five infants in China were hospitalized for varying concerns (e.g., severe gastrointestinal symptoms, or need for emergency surgery and/or treatment) and had non-respiratory symptoms as their first manifestation of COVID-19 infection [12]. The spectrum of symptoms and involvement of multiple organ systems may be due to severe acute respiratory syndrome coronavirus 2 (the virus which causes COVID-19 infection) affinity to angiotensin-converting enzyme 2 inhibitors, which are abundantly expressed in alveolar epithelial cells, as well as gastrointestinal epithelial cells, arterial and venous endothelial cells, and arterial smooth muscle cells in all organs [13-16].

To date, there have been only 10 cases reported of intussusception in children with concomitant COVID-19 infection. Seven of the cases had no preceding respiratory symptoms, and one case developed respiratory symptoms after admission. Here, we present a rare case of pediatric intussusception following COVID-19 infection.

## Case presentation

An otherwise healthy eight-month-old female with no significant medical history, born full-term, presented to the emergency department (ED) with her mother after having two bloody bowel movements along with a rash. The patient was seen in the ED two days prior where she was diagnosed with a urinary tract infection and prescribed cefdinir. During the previous visit in the ED, as a general policy of public health testing everyone for COVID-19, she tested positive for COVID-19. On examination, the patient was tearful but consolable with a heart rate of 110 beats per minute, rectal temperature of 98.9°F, respiratory rate of 24 breaths per minute, and oxygen saturation of 97% on room air.

**Figure 1:**
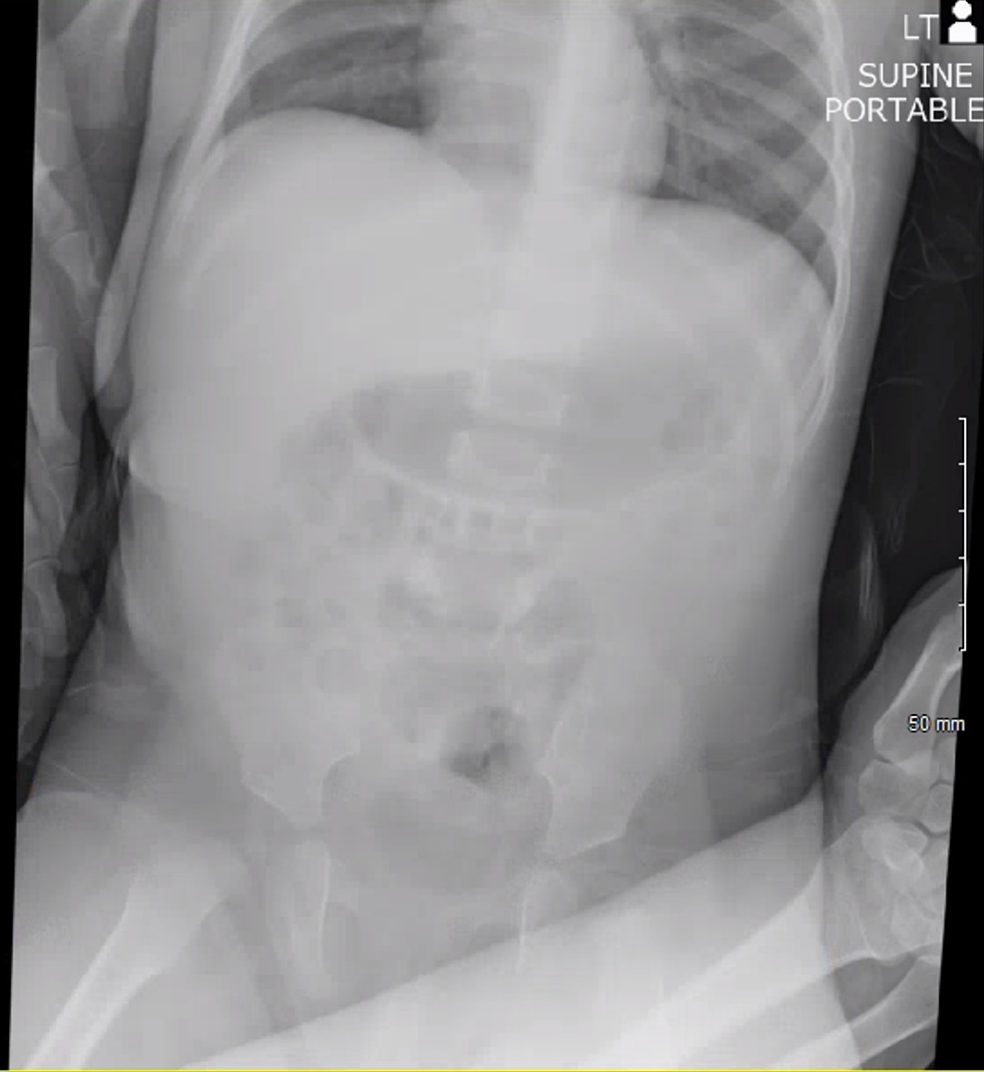
Abdominal X-ray reveals no evidence of ileus or obstruction.

**Figure 2:**
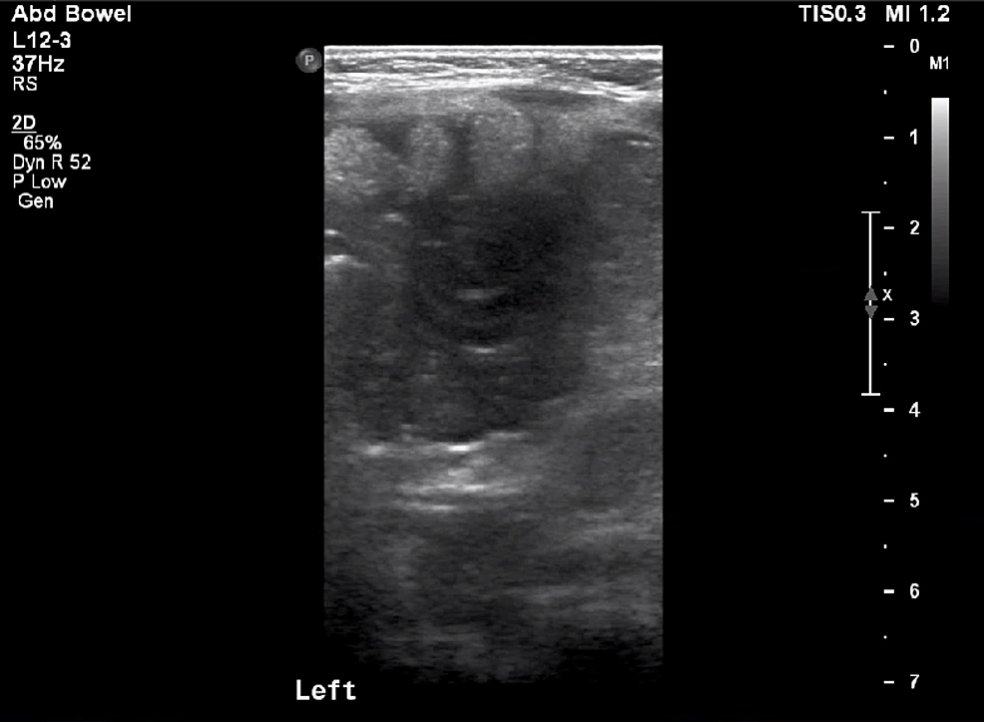
Abdominal ultrasound demonstrates “target sign” suggestive of intussusception.

The patient’s abdomen was soft, non-distended without palpable masses. Given the reported history of bloody diarrhea, a complete blood count, basic metabolic panel, abdominal X-ray, and abdominal ultrasound were ordered. Labs were significant for leukocytosis of 17,800/uL with differential showing lymphocytic predominance of 76%, signs of metabolic acidosis with bicarbonate level of 14 mEq/L, along with elevated C-reactive protein of 3.17 mg/L. No significant dilation of the small bowel, air fluid levels, or free air in the abdomen were noted on the abdominal X-ray (Figure 1). Abdominal ultrasound showed a “target sign” in the left upper quadrant (Figure 2). Given concern for intussusception, the patient was transferred to a nearby facility with higher level of capabilities and presence of pediatric gastroenterology and pediatric surgery for definitive care. A follow-up with the patient indicated an observation and bowel rest period without any surgical intervention was conducted, and after two hospital days, the patient was discharged with outpatient follow-up.

## Discussion

The most common cause of intussusception is idiopathic, accounting for 90% of cases [7]. Other causes included altered motility, infections, and anatomical factors [7]. Infectious etiologies, specifically gastroenteritis and upper respiratory infections, can lead to mesenteric lymphadenopathy and act as a lead point; in particular, adenovirus and human herpes virus-6 have both been associated with intussusception, and rotavirus vaccination has been implicated as a risk factor [11,17].

Hypertrophy of Peyer’s patches, lymphoid follicles which line the small intestine, can also occur in common viral infections and lead to intussusception [11]. Non-infectious etiologies include Celiac disease, Crohn’s disease, and neoplasia; the latter is rare in children [11]. In cases of intussusception with an identifiable etiology, a pathological lead point is a frequent cause and accounts for up to 20% of cases [18].

Several imaging modalities can assist in the diagnosis of intussusception. Plain films are useful to identify bowel obstruction and pneumoperitoneum but have low sensitivity for the detection of intussusception [19]. Ultrasound has become the modality of choice and has been shown to diagnose intussusception with 94.9% sensitivity and 99.1% specificity [20]. A positive ultrasound shows evidence of a target sign (also called doughnut sign or bull’s eye sign) which demonstrates concentric rings of mucosa, submucosa, and muscularis and represents intussusception. Computed tomography (CT) can also be used and demonstrates the appearance of bowel within the bowel, although this is sometimes an incidental finding as patients with intussusception may or may not be symptomatic [21]. CT imaging has the benefit of better characterization, including visualization of bowel edema and causative lead points, at the expense of increased cost and radiation.

The treatment of intussusception is a water-soluble contrast or air enema, given rectally, to reduce the telescoped bowel. Overall, 80% of pediatric intussusception cases are reducible with only one enema procedure, while 2.2% are not reducible after multiple attempts and require surgical intervention following enema failure [22]. When reducible, patients may be discharged home, although a more conservative approach is to observe patients in the hospital for 24 hours as the rate of recurrence is 6.5% when using a non-surgical approach [23].

A literature search revealed 10 prior cases of intussusception occurring in COVID-19-infected children that have been reported globally in the past two years. Table 1 offers a summary of all prior cases. Of note, a majority of infants (70%) presented without respiratory symptoms, and one developed respiratory symptoms following admission. Most patients were screened for COVID-19 due to hospital protocol, but one was screened due to progressively worsening respiratory status. Vomiting, cramping and intermittent abdominal pain, and red currant stool were common presenting symptoms. Infants appeared ill, showed signs of dehydration, and were described as lethargic. The majority of cases were diagnosed using ultrasound, and all cases involved infants under the age of one. Two cases ended in mortality while the remainder recovered and were doing well at follow-up.

**Table 1:** Summary of 10 prior cases of intussusception occurring in COVID-19-infected children. GI: gastrointestinal; M: male; F: female; US: ultrasound; URTI: upper respiratory tract infection; COVID-19: coronavirus disease 2019

Author	Gender, age; country	Contact history	Respiratory symptoms	GI symptoms	Other symptoms	Imaging	Diagnosis	Treatment	Outcome
Makrinioti et al., (2020) [24]	F, 10 months; China	None	Following admission	Vomiting, currant jelly stool	Paroxysmal crying	US	Intussusception, peritonitis, disseminated intravascular coagulation	Pneumatic reduction, surgical intervention	Deceased
Makrinioti et al., (2020) [24]	F, 10 months; United Kingdom	Mother with flu-like symptoms three weeks prior	Two weeks of coryzal symptoms and bilateral conjunctivitis	Bilious vomiting, currant jelly stool, lethargy	Lethargic	US	Intussusception	Pneumatic reduction, surgical intervention	Recovered
Rajalakshmi et al., (2020) [25]	M, 8 months; India	None	No	Non-bilious vomiting, currant jelly stools	Lethargic, signs of dehydration	US	Ileocolic intussusception	Pneumatic reduction	Recovered
Cai et al., (2020) [12]	F, 10 months; China	None	No	Vomiting, currant jelly stool	Paroxysmal crying, restlessness	NA	Intussusception, multiorgan failure, shock, respiratory failure	Surgical intervention	Deceased
Martínez-Castaño et al., (2020) [26]	M, 6 months; Spain	Relative with respiratory infection 12 days prior	No	Vomiting, abdominal cramps, currant jelly stool		US	Intussusception	Hydrostatic reduction	Recovered
Moazzam et al., (2020) [27]	M, 4 months; Pakistan	None	URTI one week prior	Cramping abdominal pain, red currant stool	Inconsolable crying, drawing legs to abdomthe en	US	Ileocolic intussusception	Pneumatic reduction	Recovered
Athamnah et al., (2020) [28]	M, 2.5 months; Jordan, United States	Mother with flu-like symptoms	No	Vomiting, abdominal distension, cramping abdominal pain, red currant stool	Ill-appearing, fever	US	Ileocolic intussusception	Pneumatic reduction	Recovered
Bazuaye-Ekwuyas et al., (2020) [29]	M, 9 months; United States	None	Four days of congestion, cough, sneezing; one day of fever	Vomiting, abdominal pain, decreased oral intake, blood-streaked stool	Signs of dehydration	US	Ileocolic intussusception	Hydrostatic reduction	Recovered
Mercado-Martinez et al., case 1 (2021) [30]	M, 8 months; Mexico	None	No	Non-bilious vomiting, currant jelly stool	Irritable and pale	US	Ileocolic intussusception	Surgical reduction	Recovered
Mercado-Martinez et al., case 2 (2021) [30]	F, 7 months; Mexico	None	Rhinopharyngitis one week prior	Non-bilious vomiting, currant jelly stools	Intermittent crying, fever	X-ray	Ileocolic intussusception	Surgical reduction	Recovered
Noviello et al., (2021) [5]	M, 7 months; Italy	Grandmother 10 days prior who was COVID-19-positive	No	Vomiting, abdominal pain, diarrhea, currant jelly stool	Pale, ill-appearing, signs of dehydration	US	Ileocolic intussusception	Hydrostatic reduction, surgical intervention	Recovered

## Conclusions

The number of reported cases of pediatric intussusception is increasing worldwide occurring in patients incidentally found to be COVID-19-positive. Although there are many reported gastrointestinal manifestations of COVID-19, the association with intussusception remains unclear. Given the severity of the disease process, intussusception should remain on the differential diagnosis for ill-appearing children with or without preceding viral upper respiratory symptoms and COVID-19 infection.

## References

[1] (2022). World Health Organization. Acute intussusception in infants and children: incidence, clinical representation and management: a global perspective. https://apps.who.int/iris/bitstream/handle/10665/67720/WHO_V-B_02.19_eng.pdf.

[2] Mandeville K, Chien M, Willyerd FA, Mandell G, Hostetler MA, Bulloch B (2012). Intussusception: clinical presentations and imaging characteristics. Pediatr Emerg Care.

[3] Maghrebi H, Makni A, Rhaiem R (2017). Adult intussusceptions: clinical presentation, diagnosis and therapeutic management. Int J Surg Case Rep.

[4] Azar T, Berger DL (1997). Adult intussusception. Ann Surg.

[5] Noviello C, Bollettini T, Mercedes R, Papparella A, Nobile S, Cobellis G (2021). COVID-19 can cause severe intussusception in infants: case report and literature review. Pediatr Infect Dis J.

[6] Young LL (2002). Intussusception. Case based pediatrics for medical students and residents. An Online Introductory Pediatrics Textbook.

[7] Jain S, Haydel MJ (2017). Child intussusception. https://www.ncbi.nlm.nih.gov/books/NBK431078/.

[8] Bruce J, Huh YS, Cooney DR, Karp MP, Allen JE, Jewett TC Jr (1987). Intussusception: evolution of current management. J Pediatr Gastroenterol Nutr.

[9] Blanch AJ, Perel SB, Acworth JP (2007). Paediatric intussusception: epidemiology and outcome. Emerg Med Australas.

[10] Mrak K (2014). Uncommon conditions in surgical oncology: acute abdomen caused by ileocolic intussusception. J Gastrointest Oncol.

[11] Marsicovetere P, Ivatury SJ, White B, Holubar SD (2017). Intestinal intussusception: etiology, diagnosis, and treatment. Clin Colon Rectal Surg.

[12] Cai X, Ma Y, Li S, Chen Y, Rong Z, Li W (2020). Clinical characteristics of 5 COVID-19 cases with non-respiratory symptoms as the first manifestation in children. Front Pediatr.

[13] Zhou P, Yang XL, Wang XG (2020). A pneumonia outbreak associated with a new coronavirus of probable bat origin. Nature.

[14] Richardson P, Griffin I, Tucker C (2020). Baricitinib as potential treatment for 2019-nCoV acute respiratory disease. Lancet.

[15] Hamming I, Timens W, Bulthuis ML, Lely AT, Navis G, van Goor H (2004). Tissue distribution of ACE2 protein, the functional receptor for SARS coronavirus. A first step in understanding SARS pathogenesis. J Pathol.

[16] Jia H (2016). Pulmonary angiotensin-converting enzyme 2 (ACE2) and inflammatory lung disease. Shock.

[17] Burnett E, Kabir F, Van Trang N (2020). Infectious etiologies of intussusception among children & 2 years old in 4 Asian countries. J Infect Dis.

[18] Fiegel H, Gfroerer S, Rolle U (2016). Systematic review shows that pathological lead points are important and frequent in intussusception and are not limited to infants. Acta Paediatr.

[19] Chiang JM, Lin YS (2008). Tumor spectrum of adult intussusception. J Surg Oncol.

[20] Lin-Martore M, Kornblith AE, Kohn MA, Gottlieb M (2020). Diagnostic accuracy of point-of-care ultrasound for intussusception in children presenting to the emergency department: a systematic review and meta-analysis. West J Emerg Med.

[21] Kim YH, Blake MA, Harisinghani MG, Archer-Arroyo K, Hahn PF, Pitman MB, Mueller PR (2006). Adult intestinal intussusception: CT appearances and identification of a causative lead point. Radiographics.

[22] Lee EH, Yang HR (2020). Nationwide population-based epidemiologic study on childhood intussusception in South Korea: emphasis on treatment and outcomes. Pediatr Gastroenterol Hepatol Nutr.

[23] Guo WL, Hu ZC, Tan YL, Sheng M, Wang J (2017). Risk factors for recurrent intussusception in children: a retrospective cohort study. BMJ Open.

[24] Makrinioti H, MacDonald A, Lu X (2020). Intussusception in 2 children with severe acute respiratory syndrome coronavirus-2 infection. J Pediatric Infect Dis Soc.

[25] Rajalakshmi L, Satish S, Nandhini G, Ezhilarasi S (2020). Unusual presentation of COVID-19 as intussusception. Indian J Pract Pediatr.

[26] Martínez-Castaño I, Calabuig-Barbero E, Gonzálvez-Piñera J, López-Ayala JM (2020). COVID-19 infection is a diagnostic challenge in infants with ileocecal intussusception. Pediatr Emerg Care.

[27] Moazzam Z, Salim A, Ashraf A, Jehan F, Arshad M (2020). Intussusception in an infant as a manifestation of COVID-19. J Pediatr Surg Case Rep.

[28] Athamnah MN, Masade S, Hamdallah H, Banikhaled N, Shatnawi W, Elmughrabi M, Al Azzam HS (2021). COVID-19 presenting as intussusception in infants: a case report with literature review. J Pediatr Surg Case Rep.

[29] Bazuaye-Ekwuyasi EA, Camacho AC, Saenz Rios F (2020). Intussusception in a child with COVID-19 in the USA. Emerg Radiol.

[30] Mercado-Martínez I, Arreaga-Gutiérrez FJ, Pedraza-Peña AN (2021). Intussusception and SARS-CoV-2 infection. J Pediatr Surg Case Rep.

